# Exogenous and Endogenous Stem Cells for Skeletal Regeneration

**DOI:** 10.1155/2018/2574243

**Published:** 2018-11-18

**Authors:** Heng Zhu, Bo Yu, Liu Yang

**Affiliations:** ^1^Institute of Beijing Basic Medical Sciences, Road Taiping 27, Beijing, China; ^2^Section of Restorative Dentistry, Division of Constitutive & Regenerative Sciences, UCLA School of Dentistry, Los Angeles, USA; ^3^Institute of PLA Orthopaedics & Traumatology, Xi-jing Hospital, Fourth Military Medical University, Xi'an, China

The optimized applications of stem cells for skeletal reconstruction have been gaining more and more attention in the past decades. Generally, the exogenous multipotent cells are obtained from numerous connective tissues including adipose tissues, bones, and cartilages; expanded in an appropriate cell culture system *in vitro*; and intravenously delivered into the bodies or injected into skeletal tissues. These exogenous stem cells are expected to arrive in the tissue niches, differentiate into specialized skeletal cells, and protect the skeletal tissues from harmful stimuli. However, the underlying mechanisms of the regenerative effects of exogenous stem cells and the potential influences of the *in vitro* expansion on the stem cell biological characteristics remained largely elusive, which have confined the clinical applications of stem cells for skeletal regeneration. In accordance with the therapeutic effects of exogenous stem cells in skeletal reconstruction, endogenous stem cells are known now to be involved in skeletal repair. Upon the *in vitro* and *in vivo* stimuli, these tissue-specific stem cells proliferate, migrate to the specialized tissue niches, and differentiate to reconstruct the skeletal structure and function. Also, the precise regenerative mechanisms of endogenous stem cells are incompletely understood. Therefore, more details of the regenerative effects of both exogenous stem cells and endogenous stem cells, and their underlying mechanisms would be helpful to guide us in improving stem cell-based skeletal reconstruction.

In this special issue, we first present a thorough review by W. Du et al. on the role of fibroblast growth factors in tooth development and incisor renewal. They suggest that the formation of dental tissues, as well as the development and homeostasis of the stem cells in the continuously growing mouse incisor, is mediated by multiple FGF family members. They discuss the role of FGF signaling in these mineralized tissues, trying to separate its different functions and highlighting the crosstalk between FGFs and other signaling pathways. In addition, the pivotal roles of the FGF family member in skeletal regeneration are further validated by an original article authored by L. Huang et al. They found that FGF-18 had a positive impact on chondrogenic differentiation and matrix deposition of human adipose-derived mesenchymal stem cells (ADSC). More importantly, synergistic effects of FGF-18 and TGF-*β*3 were observed on the chondrogenesis of the ADSCs *in vitro* pellet model.

Besides FGFs, platelet-derived growth factor (PDGF), a promoting factor for tissue repair, has been widely used in bone reconstruction in recent years. However, the mechanism by which PDGF regulates stem cell-based bone regeneration still remained largely unelucidated. M. Zhang et al. demonstrated that PDGF-BB increased osteogenic differentiation but inhibited adipogenic differentiation of mesenchymal stem cells (MSCs). In addition, secreted PDGF-BB significantly enhanced human umbilical vein endothelial cell migration and angiogenesis. Most importantly, they showed that PDGF-BB overexpression significantly improved MSC-mediated angiogenesis and osteogenesis *in vivo* by using a critical-sized rat calvarial defect model.

Stemness is one of the distinct features of stem cells from differentiated cells, which control the size of seed cell pool and the multipotency of seed cells for skeletal regeneration. In an original study from S. Zhang et al. in the current special issue, the researchers found that dorsal root ganglion (DRG) cells enhanced the proliferation and multipotent differentiation of MSCs. In addition, DRG cells upregulated the clone-forming ability as well as the mRNA level of Sox2, Nanog, and Oct4 of MSCs. Mechanistically, they determined that DRG cells maintain stemness of MSCs by enhancing autophagy through the AMPK/mTOR pathway.

Increasing evidence has demonstrated that skeletal regeneration is controlled by multiple factors and mechanisms. In the past decade, studies have emphasized the role of epigenetic modulation on stem cell fate. However, the study results of the ubiquitin-dependent proteolysis system in regulating bone remodeling remained controversial. Therefore, Y. Guo et al. present a thorough review on the roles of deubiquitinases in regulating differentiation and/or function of osteoblast and osteoclasts so as to reveal the multiple functions and mechanisms of deubiquitinases in bone remodeling.

In recent years, anti-inflammatory effects are considered one kind of promoting mechanisms in stem cell-based skeletal regeneration. The transplanted exogenous stem cells have been suggested for suppressing immune complications and promoting tissue repair. M. Wang et al. reviewed the advancing research on the properties of MSC-based immunomodulation and the rapidly developed clinical application of MSCs. This valuable review of MSCs provides new insight into stem cell-mediated potential treatments for tissue damage and inflammation.

While numerous stem cell-based strategies have been applied to repair skeletal defects, these techniques exhibit certain limitations including stem cell shortage and/or malfunction *in vivo*. To overcome the challenges, cell-free strategies attempt to reconstruct the damaged tissue by recruiting endogenous stem cells. W. Guo et al. compared the advantages of cell-based techniques to the cell-free counterparts and summarized potential source endogenous stem cells for skeletal regeneration. In addition, numerous important recruitment factors for meniscal regeneration were discussed. This review suggests that recruiting endogenous stem cells by cell-free techniques may play a critical role in the future of skeletal regeneration.

Furthermore, except for the modulatory mechanisms on the exogenous and endogenous stem cells, the microenvironmental factors that control the stem cell niches were also discussed in this special issue. A review from Q. Li et al. suggested that marrow adipose tissue (MAT) is a unique fat depot in the bone marrow and exhibits a close relationship with hematopoiesis and bone homeostasis. In this review, they highlight recent advancement made in MAT regarding the origin and distribution of MAT, the local interaction with bone homeostasis and hematopoietic niche, the systemic endocrine regulation of metabolism, and MAT-based strategies to enhance bone formation. They mentioned that the bone marrow niche includes a subset of skeletal stem cells capable of generating skeletal lineages and the adipocytes in the bone marrow share precursors with osteoblasts other than extramedullary adipocytes. Most importantly, they suggested that it may be a new strategy to promote skeletal regeneration by targeting MAT. Moreover, macrophages recently have been found to be an important player involved in the regulation on a stem cell niche. The strong plasticity of macrophages enables their dual function that changes the surrounding stem cell niche *in vivo*, which favors either tissue inflammation or tissue repair, which changes. X. Jia et al. investigated the potential role of macrophages during the bone regeneration process in the femurs of rats implanted with TCP. They found that TCP significantly suppresses the activation of the NF-kappa B pathway and causes a decrease in EZH1 expression. The reduction of EZH1 led to lower expressions of M1 markers and shift macrophage polarization towards the M2 phenotype. Their novel findings provide valuable insights into a new strategy that controls M2 macrophage polarization and ultimately favors a microenvironment suitable for skeletal repair.

In summary, we suggest that these outstanding original research articles and reviews may promote better understanding of exogenous and endogenous stem cell-mediated skeletal regeneration and hope the readers of this special issue will gain more insights into the advancements and challenges faced by this rapidly expanding field of medicine.

